# Development of a semi-conductor sequencing-based panel for genotyping of colon and lung cancer by the Onconetwork consortium

**DOI:** 10.1186/s12885-015-1015-5

**Published:** 2015-01-31

**Authors:** Bastiaan BJ Tops, Nicola Normanno, Henriette Kurth, Eliana Amato, Andrea Mafficini, Nora Rieber, Delphine Le Corre, Anna Maria Rachiglio, Anne Reiman, Orla Sheils, Christoph Noppen, Ludovic Lacroix, Ian A Cree, Aldo Scarpa, Marjolijn JL Ligtenberg, Pierre Laurent-Puig

**Affiliations:** 1 Department of Pathology, Radboud University Medical Centre (Radboudumc), PO box 9101, 6500 HB Nijmegen, The Netherlands; 2 Cell Biology and Biotherapy Unit, Istituto Nazionale per lo Studio e la Cura dei Tumori “Fondazione Giovanni Pascale”, IRCCS, 80131 Naples, Italy; 3 Pharmacogenomic Laboratory, CROM – Centro Ricerche Oncologiche di Mercogliano, Mercogliano, 83013 Avellino, Italy; 4 VIOLLIER, Department of Genetics/Molecular Biology, Basle, Switzerland; 5 ARC-NET Miriam Cherubini Research Centre, University of Verona, Verona, Italy; 6 Division of Theoretical Bioinformatics, German Cancer Research Center (DKFZ), 69120 Heidelberg, Germany; 7 Paris Sorbonne Cité; INSERM UMR-S775, Bases moléculaires des la réponses aux xénobiotiques, Paris, France; 8 Department of Pathology, Warwick Medical School, University Hospitals Coventry and Warwickshire, Coventry, CV2 2DX UK; 9 Department of Histopathology, The University of Dublin, Trinity College, Dublin, Ireland; 10 Department of Medical Biology and Pathology, Translational research Laboratory and Biobank (UMS3655 CNRS/US23 INSERM), INSERM Unit U981, Gustave Roussy, Villejuif, France; 11 Department of Pathology and Diagnostics, University of Verona, Verona, Italy; 12 Department of Human Genetics, Radboud University Medical Centre (Radboudumc), PO box 9101, 6500 HB Nijmegen, The Netherlands

**Keywords:** Next-generation sequencing, Semi-conductor sequencing, Colorectal cancer, Non-small cell lung cancer, Multiplex PCR, Ion Torrent

## Abstract

**Background:**

The number of predictive biomarkers that will be necessary to assess in clinical practice will increase with the availability of drugs that target specific molecular alterations. Therefore, diagnostic laboratories are confronted with new challenges: costs, turn-around-time and the amount of material required for testing will increase with the number of tests performed on a sample. Our consortium of European clinical research laboratories set out to test if semi-conductor sequencing provides a solution for these challenges.

**Methods:**

We designed a multiplex PCR targeting 87 hotspot regions in 22 genes that are of clinical interest for lung and/or colorectal cancer. The gene-panel was tested by 7 different labs in their own clinical setting using ion-semiconductor sequencing.

**Results:**

We analyzed 155 samples containing 112 previously identified mutations in the *KRAS*, *EGFR* en *BRAF* genes. Only 1 sample failed analysis due to poor quality of the DNA. All other samples were correctly genotyped for the known mutations, even as low as 2%, but also revealed other mutations. Optimization of the primers used in the multiplex PCR resulted in a uniform coverage distribution over the amplicons that allows for efficient pooling of samples in a sequencing run.

**Conclusions:**

We show that a semi-conductor based sequencing approach to stratify colon and lung cancer patients is feasible in a clinical setting.

**Electronic supplementary material:**

The online version of this article (doi:10.1186/s12885-015-1015-5) contains supplementary material, which is available to authorized users.

## Background

Although molecular mechanisms underlying carcinogenesis have been the subject of medical research for decades, only in the last few years has this information translated into the clinical setting through ‘smart’ drugs targeting specific molecular aberrations of neoplastic cells [1]. Target based agents were initially explored in unselected cohorts of cancer patients, and this approach led to failure of the majority of clinical trials with these new agents. More recently, the value of selecting patients to be treated with targeted agents on the basis of specific molecular alterations of neoplastic cells has been acknowledged [2,3]. This new therapeutic approach has led to the approval of novel drugs for molecularly selected patient populations. These agents have revolutionized the treatment of cancer patients, improving treatment outcome, while minimizing side effects.

The number of predictive biomarkers that are assessed in clinical practice is rapidly increasing with the availability of drugs that target specific molecular alterations [4]. In some tumor types, such as non-small-cell lung carcinoma (NSCLC) and colorectal carcinoma (CRC), a new classification based on the identification of specific predictive and/or prognostic molecular alterations has emerged in the past decade [5,6]. However, molecular diagnostics is faced with its own specific challenges. Costs per test, turn-around-time and the amount of material required for testing increase with the number of tests performed on a sample. In particular, the availability of tissue is limiting in NSCLC, which is often diagnosed on small histological or cytological samples yielding limited amounts of DNA [7].

Comprehensive molecular characterization of tumor tissue in clinical practice will rely on the development of high throughput technologies allowing this process to be accomplished in a cost-effective and timely manner and by using a limited amount of tissue. Two different approaches, genotyping assays and next generation sequencing (NGS) have been proposed for molecular screening [4,8]. NGS has the advantage to providing information on known and novel molecular alterations. Indeed, by using NGS-based techniques multiple genes can be sequenced simultaneously, and benchtop sequencers, like the Ion PGM™ (Life Technologies), are fast and relatively inexpensive. In this context, the ‘OncoNetwork consortium’, a European collaborative effort developed and tested the performance of a next-generation sequencing approach in a clinical setting. The advantages of a multi-lab collaboration are that methods can be quickly validated, facilitating availability, and that tests are evaluated in different laboratory settings, assessing robustness. As a proof-of-principle we chose to focus on CRC and NSCLC, two frequent tumor types for which there are known mutations associated with treatment decisions. We decided to design an Ion AmpliSeq custom gene-panel as a single multiplex PCR requiring only 10 ng of input DNA for the detection of the most frequent hot spot mutations in NSCLC and CRC. We opted for a relatively small, focused gene-panel, instead of a broader panel (like the AmpliSeq Cancer panel). Since a smaller gene-panel requires less sequence capacity more samples can be pooled in a single sequence run resulting in lower costs per sample and an increased throughput compatible with a diagnostic activity. Finally, we assessed the performance of the panel by semiconductor sequencing of 155 CRC and NSCLC formalin-fixed, paraffin-embedded (FFPE) cancer samples containing mutations previously identified by alternative genotyping methods.

## Methods

### Tumor specimens and DNA isolation

FFPE tumor samples were retrieved from the archives of the collaborating institutes and anonymised. Given the study was interpreted by all institutions (Radboud university medical centre, Istituto di ricovero e cura a carattere scientifico (IRCCS), VIOLLIER, Applied Research On Cancer Centre (ARC-NET), Institut national de la santé et de la recherche médicale (INSERM), Trinity College, Institut Gustave-Roussy and University Hospitals Coventry and Warwickshire) as a service improvement, it did not require specific research ethics committee approval as stated in the EU Clinical Trials Directive (2001/20/EC). Specific approval was obtained from the SJH/AMNCH Joint Research Ethics Committee of Trinity College, Dublin. Approval was procured for the current study as part of a wider study involving molecular analysis of a wider cohort and for research purposes that does require approval from an ethics committee. A cover letter to this effect is available.

Tissue areas for DNA extraction were micro-dissected and quantified for the percentage of neoplastic cells by a pathologist. More details regarding sample characteristics are depicted in Additional file 1: Figure S1. DNA extraction differed per institute. Most laboratories (6/7) isolated DNA using the QIAamp DNA FFPE Tissue Kit, while one lab purified DNA by overnight digestion in a proteinase K/Chelex-100 solution.

### Ion AmpliSeq™ primer design

Ion AmpliSeq™ primer pools were designed by Life Technologies (Additional file 2: Table S1 and Additional file 3: Table S2). To minimize the risk of allelic drop-out during the PCR due to primers that are not able to prime, no confirmed SNPs with a frequency >0.5% were allowed in the 5 most 3’ nucleotides. For the same reason low-frequency SNPs at other positions were kept as low as possible, with a maximum of four. Primer designs were confirmed by SNPCheck v3 analysis (www.snpcheck.net/, April 2012). The resulting oncopanel primer pool contained 87 primer pairs with an average amplicon length of 203 bp (Additional file 2: Table S1).

### AmpliSeq enrichment and Ion Torrent sequencing

Library generation was performed according to the manufacturer’s protocol. In short, 10 ng of DNA per pool was amplified in 21 cycles by PCR using the Ion AmpliSeq™ mastermix, followed by barcode and adapter ligation. Amplified products were purified with Agencourt AMPure XP beads (Beckman Coulter Genomics, High Wycombe, UK). The library was diluted to 20 pM. Emulsion PCR was performed using the Ion OneTouch™ 200 Template kit following the protocol of the Ion OneTouch™ System. Next, Ion Sphere Particles (ISPs) were recovered and enriched for template positive ISPs using Dynabeads MyOne Streptavidin C1 beads (Life Technologies) in the Ion OneTouch™ ES instrument (Life Technologies). ISP enrichment was quantified using the Qubit 2.0 fluorometer (Life Technologies). Sequencing primer and polymerase were added to the final enriched spheres before loading onto an Ion 316 chip according to the Ion PGM™ 200 sequencing kit protocol.

### Data analysis

Sequence data were directly uploaded from the Ion Torrent Suite Software to the IonReporter™ Software version 1.2 and data were analyzed using a pre-configured workflow specific for detecting low frequency somatic variants. The allele frequency cut-off was set at 0.04. In addition, the data were analyzed in parallel using a ‘hotspot bed file’ containing hotspot mutations in the regions of our gene-panel derived from the COSMIC database. The allele frequency cut-off for hotspot analysis was set at 0.02. These cutoffs were chosen following a preliminary phase in which, starting from samples with known sequence, the best bioinformatic algorithm ensuring the highest sensitivity and specificity was identified. The workflow includes mapping statistics, measurements of the quality of all reads, on and off target statistics, SNP and INDEL calling and comprehensive annotation using public databases (dbSNP version 137, COSMIC version 60, OMIM version 08012012, Gene Ontology version 1.218). The variant caller algorithm was used and variants that were identified for each sample per lab in each phase were further filtered. We applied quality filtering by discarding reads with a quality score <40 and checking against strand bias. We filtered against variants with a variant allele count less than 10, variants that showed up in every sample of one lab with the same allele frequency, the synonymous and non-coding variants and variants that were known as polymorphisms or with a Minor Allele Frequency (MAF) greater than 35%. As a result, a variant knowledgebase was generated with the variants detected in those samples. A specific workflow for this panel was configured in the online Ion Reporter™ Software and was used to perform analysis for data generated using the Colon/Lung panel.

## Results

### Panel design and performance

The panel was designed in order to contain well-known predictive markers in the receptor tyrosine kinase (RTK) pathway, such as mutations of the *EGFR* (for NSCLC) and *KRAS* (for NSCLC and CRC) genes, but also included genes that might serve as targets in the near future or have a prognostic relevance, like *BRAF* [9,10], *AKT1* [11], *DDR2* [12,13] and *ERBB2* [14-16] (Table 1). Selection of the gene regions was based on their mutation frequencies, based on the COSMIC database (Table 1). In particular, the entire gene-panel targets 87 hotspot regions for the following 22 genes: RTKs (*ALK*, *EGFR, ERBB2, ERBB4, FGFR1, FGFR2, FGFR3, MET, DDR2*); RTK signaling genes (*KRAS, PIK3CA, BRAF, AKT1, PTEN, NRAS, MAP2K1, STK11*); and other well known cancer-related genes (*NOTCH1, CTNNB1, SMAD4, FBXW7, TP53*).

**Table 1:** **Mutation frequency of genes for which hotspot regions are present in the gene-panel**

Gene	Chr	RefSeq	Function	% Mutated CRC^#^	% Mutated lung cancer^#^
*AKT1*	14	NM_005163.2	Oncogene	0.64	0.42
*ALK*	2	NM_004304.3	Oncogene	2.74	5.25
*BRAF*	7	NM_004333.4	Oncogene	12.19	2.46
*CTNNB1*	3	NM_001904.3	Oncogene	4.61	3.13
*DDR2*	1	NM_006182.2	Oncogene	0	0.21
*EGFR*	7	NM_005228.3	Oncogene	2.75	26.71
*ERBB2*	17	NM_004448.2	Oncogene	1.48	1.80
*ERBB4*	2	NM_005235.2	Oncogene	3.84	8.35
*FGFR1*	8	NM_023110.2	Oncogene	0.55	1.62
*FGFR2*	10	NM_000141.4	Oncogene	0.42	1.68
*FGFR3*	4	NM_000142.4	Oncogene	0.53	0.92
*KRAS*	12	NM_004985.3	Oncogene	34.51	16.23
*MAP2K1*	15	NM_002755.3	Oncogene	0.15	1.14
*MET*	7	NM_001127500.1	Oncogene	0.73	3.03
*NOTCH1*	9	NM_017617.3	Oncogene	0.13	3.44
*NRAS*	1	NM_002524.3	Oncogene	3.37	0.88
*PIK3CA*	3	NM_006218.2	Oncogene	11.90	4.08
*FBXW7*	4	NM_0033632.2	Tumor suppressor	6.83	3.00
*PTEN*	10	NM_000314.4	Tumor suppressor	3.86	3.59
*SMAD4*	18	NM_005359.5	Tumor suppressor	7.89	2.68
*STK11*	19	NM_000455.4	Tumor suppressor	1.28	8.40
*TP53*	17	NM_000546.5	Tumor suppressor	41.75	37.64

^#^Data was extracted from the COSMIC database (http://cancer.sanger.ac.uk/cancergenome/projects/cosmic/) February 4^th^ 2013.

Dilution experiments with an artificial control sample containing 10 hotspot mutations in the *BRAF, EGFR, KRAS, NRAS* and *PIK3CA* genes (Horizon Diagnostics) demonstrated that hotspot mutations could be confidentially identified as low as 2% mutant alleles provided that the coverage was >500× (results not shown). Preliminary testing of the lung/colon cancer primer pool showed that up to 5 samples could be pooled on a 316 chip (Ion PGM™ Sequencer) with a minimal average read-depth of 500× (results not shown).

### Experimental design

**Figure 1:**
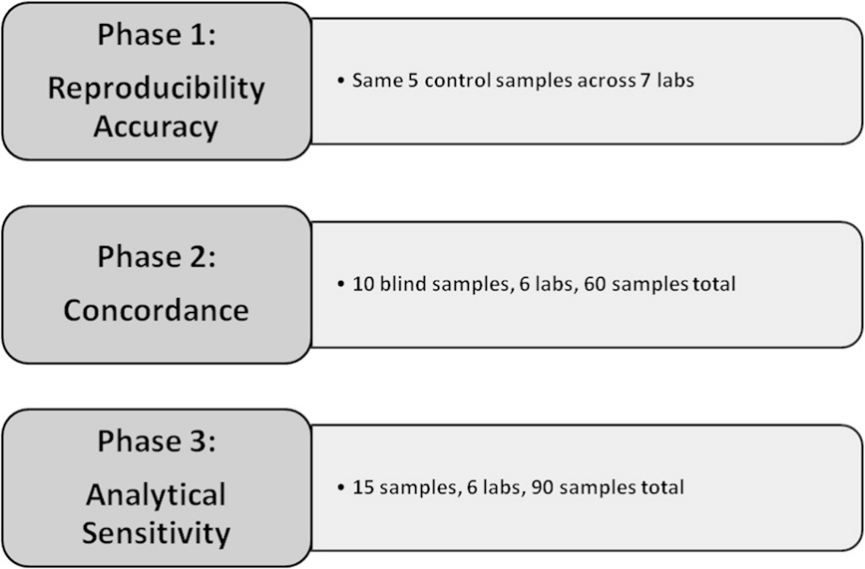
**Schematic scheme of the 3 phases to assess the performance of the gene-panel.**

The above described panel, from now on called version 1 (v1), was used to test 155 FFPE tissue samples that contained mutations previously identified by other methods. In particular, the performance of the panel was assessed in three phases (Figure 1). The first phase aimed at setting up the AmpliSeq protocols, workflow and data analysis and to define accuracy and precision of the panel. To this end, 7 consortia labs tested 5 control samples in an inter-laboratory ‘ring-trial’, i.e. 2 AcroMetrix® controls (FFPE colon cancer cell lines, A12 and A13), 2 FFPE xenograft colon tumours (X23 and X32) and one FFPE lung sample (L1). All labs correctly identified in the control samples the mutations previously detected by Sanger sequencing (Table 2).

**Table 2:** **Variants identified in the 5 control samples**

Sample	Known mutations	Mutations identified by NGS^‡^	Allele frequency^#^
L1	*KRAS*: p.Gly12Cys	*KRAS*: p.Gly12Cys	0.44 - 0.54
A12	*KRAS*: p.Gly12Ala	*KRAS*: p.Gly12Ala	0.66 - 0.67
		*FBXW7*: p.His460Tyr	0.46 - 0.49
		*TP53*: p.Ala159Asp	0.74 - 0.75
A13	*KRAS*: Gly13Asp	*KRAS*: Gly13Asp	0.50 - 0.56
		*PIK3CA*: p.Asp549Asn	0.43 - 0.46
		*DDR2*: p.Thr98Ala	0.47 - 0.56
		*FGFR1*: p.Ala268Ser	0.50 - 0.66
		*NOTCH1*: p.Pro1581Leu	0.48 - 0.54
X23	*KRAS*: p.Gly12Asp	*KRAS*: p.Gly12Asp	0.65 - 0.75
	*PIK3CA*: p.Glu545Lys	*PIK3CA*: p.Glu545Lys	0.40 - 0.51
X32	*KRAS*: p.Gly12Asp	*KRAS*: p.Gly12Asp	0.54 - 0.69
	*PIK3CA*: p.Glu542Lys	*PIK3CA*: p.Glu542Lys	0.44 - 0.46
	*FBXW7*: p.Arg465His	*FBXW7*: p.Arg465His	0.46 - 0.55
	*TP53*: p.Gly244Asp	*TP53*: p.Gly244Asp	0.32 - 0.35

^**‡**^Newly identified variants were verified by conventional Sanger sequencing.

^**#**^Indicated is the range of the allele frequencies over the different laboratories.

By using the panel, all 7 labs also identified 6 new variants in the two AcroMetrix® control samples (Table 2). To confirm that these new variants are not sequencing artifacts, we re-analyzed these positions by conventional Sanger sequencing. All 6 new variants were confirmed suggesting the specificity of our workflow (data not shown). The two xenograft samples were not analyzed for new variants since off-target amplification of mouse DNA hampered data analysis.

In the second phase, we designed a ring-trial in which 6 labs selected 10 FFPE specimens (5 lung and 5 colon carcinoma FFPE samples) previously tested in a diagnostic setting (for sample characteristics see Additional file 1: Figure S1A). These samples were tested, in the blind, by a second consortium lab. In total, the selected 60 samples contained 47 previously identified mutations (42 missense and 5 indels) in the *KRAS*, *EGFR*, *BRAF* and *CTNNB1* genes (Table 3). All previously detected mutations were detected using our oncopanel v1 and the IonReporter variant caller.

**Table 3:** **Known mutations present in the 60 samples that were analyzed in the blind during phase 2 of the panel validation**

Mutation	Unique samples	Identified
BRAF: p.Val600Glu	3	Yes
CTNNB1: p.Thr41Ile	1	Yes
CTNNB1: p.Asp32Asn	1	Yes
EGFR: p.Glu746_Arg748del	1	Yes
EGFR: p.Glu746_Ala750del	4	Yes
EGFR: p.Glu746_Ser752del	1	Yes
EGFR: p.Leu858Arg	5	Yes
KRAS: p.Gln61Arg	1	Yes
KRAS: p.Gly12Arg	2	Yes
KRAS: p.Gly12Cys	5	Yes
KRAS: p.Gly12Asp	8	Yes
KRAS: p.Gly12Ala	2	Yes
KRAS: p.Gly12Val	7	Yes
KRAS: P.Gly13Cys	1	Yes
KRAS: p.Gly13Asp	5	Yes

**Figure 2:**
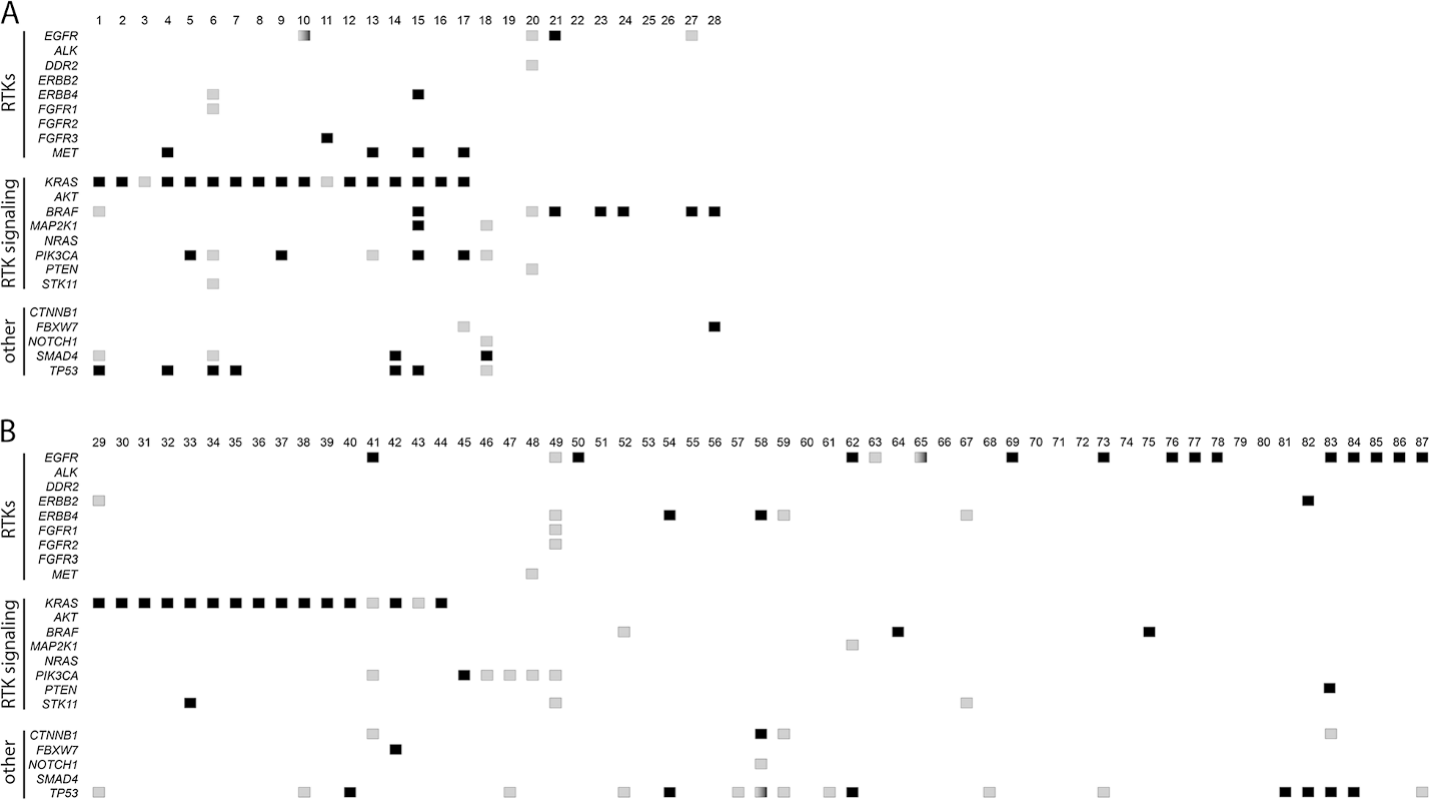
**Schematic representation of the results of phase 3 of the panel validation.** Indicated are the variants per sample identified in the 28 CRC **(A)** and 59 NSCLC **(B)** samples. Variant frequencies were extrapolated to 100% neoplastic cells and/or related to other variants present in the sample (intra-sample comparison). Variants in black correspond to ‘driver’ mutations (present in >35% of neoplastic alleles), while variants in grey are present in only a minority of the neoplastic cells (<35% of neoplastic alleles or an allele frequency 0.5x that of the driver mutation present in the sample). Genes containing >1 variant in different frequencies are indicated in grey/black boxes.

In the third and final phase, each lab selected 15 in-house samples representative of the type of samples encountered in a diagnostic clinical setting and that are problematic to analyze due to paucity of material, such as small biopsies or cytological material, low percentage of neoplastic cells and poor DNA quality (for sample characteristics see Additional file 1: Figure S1B). In total 29 CRC and 61 NSCLC samples were selected. In these samples, 56 mutations in the *KRAS*, *EGFR* and *BRAF* genes (46 missense and 10 indels) were previously identified in 54 unique samples using different methods (Additional file 4: Table S3). DNA of 1 sample failed to amplify due to low quality (technical failure 1.1%). All other samples could be analyzed and all known variants were identified. Two additional samples were excluded from further in-depth analyses (2.2%), since these samples contained a high number of variants (>15) with an allele frequency of 4-7%, probably due to (over) fixation with formalin [17]. In the remaining 87 assessable samples, we identified 92 new variants in regions that were not analyzed with the previously used alternative method (mutations and low frequency SNPs with a MAF <0.04). Most of these new variants were identified in the RTK-signaling genes *BRAF*, *EGFR*, *KRAS* and *PIK3CA* (24 mutations) and in *TP53* (30 mutations) (Figure 2 and Additional file 5: Table S4). Furthermore, when taking the percentage of neoplastic cells in individual samples and the allelic frequency of the variants into account, in contrast to most mutations in *KRAS*, *EGFR* and *BRAF*, a large proportion of the newly identified mutations appear to be present in only a subset of tumor cells (Figure 2).

Thus, combining the data of all experiments, we analyzed 155 unique samples containing 112 previously identified mutations. All specimens were correctly genotyped, except one that could not be analyzed due to poor quality of the DNA, demonstrating excellent performance of the gene panel.

### Sequencing efficiency and panel re-design

**Figure 3:**
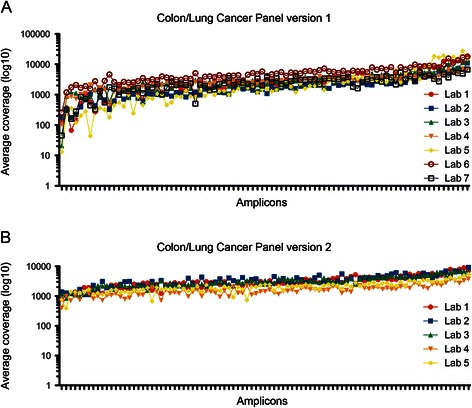
**Average coverage distribution over the individual amplicons.** The coverage distribution plot over the individual amplicons in gene-panel v1 **(A)** and v2 **(B)**. For panel v1 5 samples are pooled per 316 chip; with panel v2 8 samples are pooled per 316 chip **(B)**. Note the logarithmic scale of the y-axis.

While the level of concordance for mutational analysis using this panel compared to classical Sanger sequencing is excellent, we could ‘only’ multiplex 5 samples on a 316 chip to reach a minimal read depth of 500× per amplicon. To determine if we could reduce the cost per sample by multiplexing more samples per chip, we assessed the sequencing efficiency by determining the read distribution over the individual amplicons. While the average coverage over the amplicons was reproducible among different labs and runs (Figure 3A and not shown), the difference between the lowest and highest covered amplicons was substantial (average coverage of 3274 ± 2470 reads). While this in itself is not a problem, the available sequencing capacity of the chip is not efficiently used. Since an improved primer design algorithm was introduced by Life Technologies after the design of panel v1, the panel primers were re-designed to attempt to optimize the read distribution over the amplicons (Additional file 3: Table S2). Moreover, the amplicons were designed to be smaller to facilitate the amplification of DNA extracted from FFPE specimens (91 amplicons, average size 158 bp). As shown in figure 3B, the read distribution of v2 over the individual amplicons was superior to v1 (lowest and highest covered amplicons within 10 fold range). Due to the improved read distribution, up to 8 samples could be pooled on a 316 chip with an average read depth of 2751 ± 1107 reads.

To check the performance of the panel, the 5 control samples of phase 1 were-resequenced by all 7 laboratories, and the expected variants as well as the new variants identified in the two Accrometrix control samples were detected using panel v2. In addition, the new panel was used by 5 labs to re-sequence their 15 in-house samples (75 samples in total) previously tested with panel v1, and 6 labs also tested 5 samples not previously analyzed (not all labs were able to participate). All mutations previously detected with panel v1 were again identified using panel v2 (data not shown), demonstrating that this panel performs as well as v1 for these mutations. However, since 8 samples can be multiplexed on a 316 chip with panel v2, instead of 5 samples using v1, the sequencing costs per sample are significantly reduced.

## Discussion

Specification of the molecular class of NSCLC and CRC is mandatory to optimize personalized medicine for these diseases. However, a comprehensive molecular characterization of the tumor is faced with a number of challenges, including cost per test, turn-around-time and the limited amount of material available for genotyping. NGS-based approaches can overcome these challenges by providing in a single analysis information on tumor inter- and intra-heterogeneity that can be relevant in a clinical setting. In this respect, a number of NGS panels for tumor profiling have become commercially available. However, these panels lack of validation in clinical samples and their inter-laboratory reproducibility has not been shown. In this study we propose a novel model of collaboration between clinical research laboratories and NGS-companies that led to the development of a tool that is ready for application in a clinical setting. Indeed, the validation of panels through a consortium of academic institutions represents a novel approach to speed the development of new diagnostic tools, being able to provide information on a significant number of clinical specimens and on inter-laboratory reproducibility.

We must acknowledge that there are still several issues that need to be addressed to implement NGS panels in the clinic. Our panel included 22 genes, although the approved predictive biomarkers in colon and lung cancer are quite few (*KRAS, NRAS, EGFR, ALK*). Nevertheless, in many cancer comprehensive centers clinical trials with new drugs are open and molecular pathologists are requested to screen for a number of biomarkers that are included in the Colon and Lung cancer panel that we developed. For example, among the 61 difficult NSCLC samples analyzed in the third phase, we identified 18 *EGFR*, 7 *PIK3CA* and 3 *BRAF* mutants. Since drugs targeting these latter molecular alterations are in clinical trials, the availability of this information might significantly improve the possibility of these patients to receive a personalized therapy [5]. It is possible that novel biomarkers will be discovered in the future. In this respect, one advantage of this panel is that it can be easily adapted by adding new amplicons/genes of interest. Of course, a re-validation on a minimum number of samples is recommended for any modification of the panel.

The performance of a new test is a key element in evaluating the possibility to introduce it in clinical diagnostics. In this regard, we chose to analyze our data with an allele frequency cut-off of 4% for all variants and 2% for known hotspot mutations corresponding to 5-10% neoplastic cells carrying heterozygous mutations. This estimate is in agreement with a previous study that assessed somatic mutations in FFPE material by using semiconductor-based massive parallel sequencing [18]. The allele frequency cut-off required for the detection of somatic mutations is debatable, but samples with <10% neoplastic cells are also difficult to diagnose by classical histomorphological methods. Using this allele-frequency cut-off we tested 155 clinical FFPE samples that were previously analyzed by conventional methods. A substantial number of these specimens were specifically selected based on the fact that they were difficult to analyze with routine techniques. Even with this bias in sample selection we were still able to correctly analyze 154 of the 155 samples. Although our sample set was selected for certain mutations, our specimens contained variants that represent the full spectrum of mutations that are identified in NSCLC and CRC specimens. These include a variety of indel and missense mutations of which also hotspot mutations present in only 2% of the alleles were readily detected. Other studies have previously suggested that semiconductor-based sequencing of FFPE tissue is indeed feasible [18-20]. However, this is the first study that evaluated a so large collection of FFPE samples.

Other mutation-detection techniques, like quantitative PCR (qPCR) or high resolution melting (HRM), can also reach this level of sensitivity, but lack information regarding the allele-frequency of the mutation. For example, EGFR mutations showed allelic frequencies ranging between 20% and 210% (amplification), after normalization for the percentage of neoplastic cells. This information could be clinically relevant in the near future as suggested by a report showing that the duration of the response to target based agents might be related to the relative content of mutant alleles in the tumor [21]. A quantitative assessment of the different mutant clones in polyclonal tumors will also be useful for treatment decision, as recently shown for EGFR sensitizing and resistance mutations in NSCLC [22]. In addition, genotyping techniques can only provide information on known mutations whereas sequencing-based methods can also detect novel and rare mutations. Some of the new variants we identified might not be true somatic mutations. Variants with high allelic frequencies could also represent infrequent SNPs, while especially non-hotspot variants with low allelic frequencies could be false positive calls. These are difficult to confirm however with standard methods like Sanger sequencing. It must be emphasized that during this project we used IonReporter Software version 1.2, while currently version 1.6 is available with improved mutation calling, especially in homopolymer regions.

Conventional methods, like Sanger sequencing or qPCR, are singleplex tests and, as the number of targets increases, the amount of DNA required for multiple tests is a limiting factor. This can be problematic especially in lung cancer, since molecular analyses are often performed on fine needle aspiration (FNA) biopsies or cytology samples yielding limited amounts of DNA. Our single multiplex PCR, consisting of 87 amplicons targeting 22 genes and requiring as little as 10 ng of input DNA, overcomes this problem. Analyzing a similar number of amplicons by conventional methods would require 500–1000 ng of DNA, clearly demonstrating the advantage of NGS.

Turn-around-time (TAT) is an important variable to take into account in clinical diagnostics. We opted for the IonPGM in our institutes since benchtop sequencers, like the Ion PGM or the MiSeq only need hours to generate sequence data. In combination with a sample preparation that is fast, like the Ion AmpliSeq™ gene-panel presented here, the TAT from DNA isolation to results is between 48 and 72 hours. While this may not be as fast as some conventional methods, it is adequate for most routine clinical applications.

An additional important consideration is cost. NGS in general is cheap if costs are calculated per sequenced base. However, clinical diagnostic laboratories do not need to sequence hundreds of genes (yet), and neither can they afford to batch numerous samples because of the short TAT needed for clinical specimens. It is therefore important that the sequencing capacity of the NGS platform is flexible and can be adjusted based on the requirements per experiment (e.g. the number of clinical specimens may vary from day to day). A second consideration is that the available sequence capacity on the chip is efficiently used. Since our custom panel specifically investigates genetic aberrations of interest for CRC and NSCLC, the required sequencing capacity for our panel is smaller compared to commercial panels that have been designed for a broad spectrum of cancers. We estimate the bill of materials costs between €130 and €175 per sample (excluding sales tax, depreciation and overhead costs) using the re-designed panel. While this may be higher compared to a single conventional diagnostic test, it is actually a lot cheaper considering that the NGS approach replaces multiple conventional tests (Additional file 6: Table S5 and [18]).

## Conclusion

The rapidly changing landscape in the field of tumor molecular characterization requires the analysis of an increasing amount of targets. With the availability of fast bench top sequencers such as the Ion Torrent PGM, massive parallel sequencing becomes available for clinical laboratories. Here we showed that the development and validation of new tests is feasible with a multi-lab effort, and believe it will facilitate the introduction of these tests into clinical settings. We also demonstrated that implementation of massive parallel sequencing using dedicated gene-panels can be fast, cost-effective and accurate. More importantly, this novel approach might facilitate the adoption of a molecular classification of lung and colon carcinoma in clinical research, thus improving the possibility to identify tumors carrying actionable molecular alterations. Finally, we want to highlight that organization of consortia similar to the one that we established in this study might represent a novel approach to have a rapid, un-biased validation among academic Institutions of new testing methods in a clinical scenario.

## Additional files

Additional file 1: Figure S1. Depicted are the characteristic s for the samples used in phase 2 (A) and phase 3 (B) of the study. Indicated are the neoplastic cell content of the samples (information provided by 6/7 labs), the type of tissue (biopsy or resection), origin of tumor tissue (primary or metastasis), if the tumor tissue was micro-dissected, the method previously used to determine mutation-status and for the samples in phase 3 the reason for inclusion.
Additional file 2: Table S1. Gene-panel version 1.
Additional file 3: Table S2. Gene-panel version 2.
Additional file 4: Table S3. Known mutations present in the 90 samples that were analyzed during phase 3 of the panel validation.
Additional file 5: Table S4. All variants identified in the 90 samples that were analyzed during phase 3 of the panel validation. Indicated are the identified variants and the allele frequency.
Additional file 6: Table S5. Approximate indication of hands on time and costs of Sanger sequencing and Next Generation Sequencing of the genetic regions covered by the described gene panel.
