# Supplementary figures and images for: Development of a semi-conductor sequencing-based panel for genotyping of colon and lung cancer by the Onconetwork consortium
Source: BMC Cancer. 2015 Jan 31;15:26. doi: 10.1186/s12885-015-1015-5 (PMC4318366; doi:10.1186/s12885-015-1015-5)

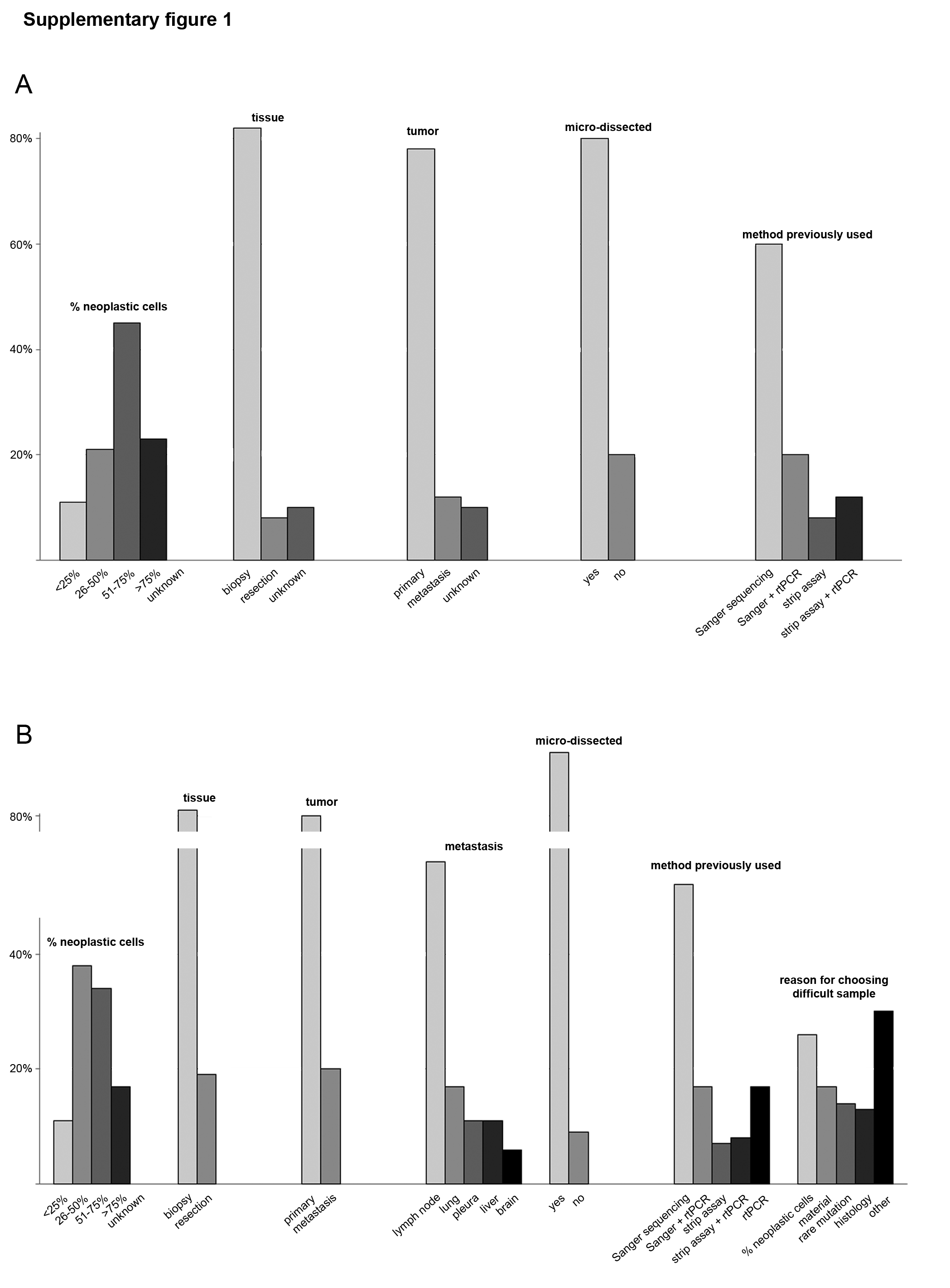

Supplement: Additional file 1: Figure S1. — Depicted are the characteristic s for the samples used in phase 2 (A) and phase 3 (B) of the study. Indicated are the neoplastic cell content of the samples (information provided by 6/7 labs), the type of tissue (biopsy or resection), origin of tumor tissue (primary or metastasis), if the tumor tissue was micro-dissected, the method previously used to determine mutation-status and for the samples in phase 3 the reason for inclusion. [file 12885_2015_1015_MOESM1_ESM.jpeg]
