# Supplementary material for: Development of a semi-conductor sequencing-based panel for genotyping of colon and lung cancer by the Onconetwork consortium
Source: BMC Cancer. 2015 Jan 31;15:26. doi: 10.1186/s12885-015-1015-5 (PMC4318366; doi:10.1186/s12885-015-1015-5)
Supplement: Additional file 2: Table S1. — Gene-panel version 1. [file 12885_2015_1015_MOESM2_ESM.docx]

**Supplementary table 1.** Gene-panel version 1.

| **Gene** | **Chr** | **RefSeq^#^** | **Oncogene / Tumor suppressor gene** | | **Codons** |
| --- | --- | --- | --- | --- | --- |
| *AKT1* | 14 | NM_005163.2 | Oncogene |  |  |
| exon3 | CCCCAAATCTGAATCCCGAGA | | GGGTCTGACGGGTAGAGTGT | | 16-59 |
| *ALK* | 2 | NM_004304.3 | Oncogene |  |  |
| exon22 | CGATCTGTTAGAAACCTCTCCAGGT | | CTTGGCTTGCGGACTCTGTA | | 1151-1172 |
| exon23 | CCTGTCCTTGGCACAACAACT | | CCAGACTCAGCTCAGTTAATTTTGGT | | 1172-1215 |
| *BRAF* | 7 | NM_004333.4 | Oncogene |  |  |
| exon11 | TGACTTGTCACAATGTCACCACAT | | TTTTCTGTTTGGCTTGACTTGACTTTTT | | 439-478 |
| exon15 | AGCATCTCAGGGCCAAAAATTTA | | GCTCTGATAGGAAAATGAGATCTACTG | | 581-620 |
| *CTNNB1* | 3 | NM_001904.3 | Oncogene |  |  |
| exon3 | TGTTTCGTATTTATAGCTGATTTGATGGA | | CCTCTTCCTCAGGATTGCCTTT | | 10-48 |
| *DDR2* | 1 | NM_006182.2 | Oncogene |  |  |
| exon5 | GAAAACTGTGGCAAGAACCCAAA | | CTGCAGAAACTCCTTCAGGTCA | | 62-81 |
| exon8 | GTGTGGTTAACTCAGATTTCTCTCTCC | | ATTCCTGATGCGGTCAAATTCAAAC | | 224-271 |
| exon12 | ACATGCCTTTCTCCTTGCTCTTC | | GAGCAAATTCTGGGAGTTTTCGT | | 432-487 |
| exon13 | CATGTTTTAGCCCTCCTCTCAGA | | CACTGAGTATGTGTTGCCTCCT | | 502-532 |
| exon14 | TGATCATTTTGCCTGAGTTGTAAGAGTT | | TCTGCTCGGAGCATTTTCACA | | 577-606 |
| exon15 | GGTGTGCATTCTTCTCTCTCTTGA | | CGTGGCGGGAAAGAAACTGAT | | 619-662 |
| exon17 | GGTGTTGTTGTGCACAGGTTAT | | CAGTTCTTACCTGCCTCCCTTG | | 762-807 |
| *EGFR* | 7 | NM_005228.3 | Oncogene |  |  |
| exon12 | TCAAGGAGATAAGTGATGGAGATGTGAT | | ACAAATAAAGGACCCATTAGAACCAACTC | | 463-500 |
| exon18 | TGAGGTGACCCTTGTCTCTGT | | CTCCCCACCAGACCATGAG | | 688-728 |
| exon19 | GCATGTGGCACCATCTCACAA | | CATGAGAAAAGGTGGGCCTGA | | 729-761 |
| exon20 | CATGCGAAGCCACACTGAC | | AGCAGGTACTGGGAGCCAATAT | | 762-807 |
| exon21 | CCTCACAGCAGGGTCTTCTC | | AGGAAAATGCTGGCTGACCTAAA | | 824-875 |
| *ERBB2* | 17 | NM_004448.2 | Oncogene |  |  |
| exon19 | AGGGCATCTGGATCCCTGAT | | ATATCTCCCCAAACCCCAATGAAG | | 743-769 |
| exon20 | GGGTGTGTGGTCTCCCATAC | | ATCTGCATACACCAGTTCAGCA | | 770-821 |
| exon21 | CCTCCCAGAAGGTCTACATGG | | CTCTGCTCCTTGGTCCTTCA | | 832-883 |
| *ERBB4* | 2 | NM_005235.2 | Oncogene |  |  |
| exon3 | CTGATATTTAAATGCCTTAGAGTGTTCCT | | GAGGATCGATATGCCTTGGCAATA | | 120-141 |
| exon4 | GCCATTTGTTCTGCCATATATAACTGA | | CATTCATTGGCAAGATATTGTTCGGA | | 169-185 |
| exon6 | AAAGTGGCTAAAGTTGATCTGATTGTA | | CGGTGTGTGCAGAACAATGTG | | 219-247 |
| exon7 | TCTGTTACTTACGTGGACATTTCTTGAC | | TGACAGTGGAGCATGTGTTACTC | | 261-289 |
| exon8 | ACTAAATAATCTGAGCTACCACTCACCT | | TGTTTTGAGCTTGTTTGCTGAATGTTAA | | 295-332 |
| exon9 | ACTGACCCATGAATACCAGTGACT | | TGAGTCTTGTTTCTACAGCTTGTGATG | | 336-369 |
| exon15 | CACATACCAGGTGAGCCCTTG | | CATTTCAGGGTCCTGACAACTGT | | 578-624 |
| exon23 | GCAGTCTTACATTTGACCATGACCAT | | GGAGTTACTATATGGGAACTGATGACCT | | 916-950 |
| *FGFR1* | 8 | NM_023110.2 | Oncogene |  |  |
| exon4 | CCCTCCCCTCTTAAACCCAAT | | GGAACTGCACTAGCCTTGGTG | | 120-150 |
| exon7 | CCCATTCACCTCGATGTGCTTT | | GTGAGCCCACCCCTCTTTAG | | 249-289 |
| *FGFR2* | 10 | NM_000141.4 | Oncogene |  |  |
| exon7 | AGAATCATCCTCTCTCAACTCCAACA | | ACGTAGAGTTTGTCTGCAAGGTTT | | 281-313 |
| exon9 | GGGATACGTTTGGTCAGCTTGT | | CCTGCTTATCTGTTCCTCCTCCT | | 362-415 |
| exon12 | TGGAAGCCCAGCCATTTCTAAA | | TTCATAGATGATGCCACAGAGAAAGAC | | 528-558 |
| *FGFR3* | 4 | NM_000142.4 | Oncogene |  |  |
| exon7 | GCAGACGTACACGCTGGA | | CTGTGCGTCACTGTACACCTT | | 238-283 |
| exon9 | CAACGCCCATGTCTTTGCAG | | CTACTTTCTGTTACCTGTCGCTTGA | | 359-418 |
| exon14 | CCCCTTCCCCAGTGCATC | | TGTGGGAAGGCGGTGTT | | 615-653 |
| exon16 | GGTGTCTGTCCTGGGAGTCT | | GATGCCACTCACAGGTCGT | | 678-720 |
| exon18 | GGCGCCTTTCGAGCAGTA | | ATCTGCACTGAGTCTCATGCC | | 771-806 |
| *KRAS* | 12 | NM_004985.3 | Oncogene |  |  |
| exon2 | TGTTGGATCATATTCGTCCACAAAATGA | | AGTGTATTAACCTTATGTGTGACATGTT | | 1-26 |
| exon3 | TTCAATTTAAACCCACCTATAATGGTGA | | TTTCTCCCTTCTCAGGATTCCTACA | | 41-93 |
| exon4 | CAGATCTGTATTTATTTCAGTGTTACTTACCT | | GACTCTGAAGATGTACCTATGGTCCTA | | 114-149 |
| *MAP2K1* | 15 | NM_002755.3 | Oncogene |  |  |
| exon2 | GTATTGACTTGTGCTCCCCACTT | | CCAGAAGGCTTGTGGGAGAC | | 28-84 |
| exon3 | AAACCTCTCTTTCTTCCACCTTTCTC | | CCCAACTCTTAAGGCCATTGCT | | 98-146 |
| exon4 | CACAGCCGAAAGTTATCACTTGAAA | | CTCTTGTCCATTTCTTACCGACTCAT | | 147-172 |
| exon6 | TCCCCAATCTACCTGTGTCAGT | | CCTGACCGTACAAGAAGCTCAAG | | 190-231 |
| *MET* | 7 | NM_001127500.1 | Oncogene |  |  |
| exon2_1 | CCTGCCAGCGACATGTCTTT | | GCAATGGATGATCTGGGAAATAAGAAGA | | 147-202 |
| exon2_2 | GGAGCCAGCCTGAATGATGA | | GGGAACTGATGTGACTTACCCT | | 341-399 |
| exon14 | CTCTCTGTTTTAAGATCTGGGCAGT | | ACAACCCACTGAGGTATATGTATAGGT | | 985-1028 |
| exon16 | TGTTACGCAGTGCTAACCAAGT | | TGCCACTTACTGTTCAAGGATTTCA | | 1105-1126 |
| exon19 | TGTCCTTTCTGTAGGCTGGATGA | | AGAGGAGAAACTCAGAGATAACCAATACA | | 1233-1284 |
| *NOTCH1* | 9 | NM_017617.3 | Oncogene |  |  |
| exon26 | TCCTCGCGGCCGTAGTA | | GGGACGGGCTGGACTGT | | 1562-1618 |
| exon27 | GCACGGCCTCGATCTTGTA | | CCCAGCTCCATCGTCTACCT | | 1679-1715 |
| *NRAS* | 1 | NM_002524.3 | Oncogene |  |  |
| exon2 | CGACAAGTGAGAGACAGGATCA | | CTGTAGATGTGGCTCGCCAAT | | 1-37 |
| exon3 | AATAATGCTCCTAGTACCTGTAGAGGTT | | CCACACCCCCAGGATTCTTAC | | 41-93 |
| *PIK3CA* | 3 | NM_006218.2 | Oncogene |  |  |
| exon10 | CAGAGTAACAGACTAGCTAGAGACAATGA | | CATTTTAGCACTTACCTGTGACTCCAT | | 523-550 |
| exon14 | ACGATTCTTTTAGATCTGAGATGCACAA | | TCTCAAACACAAACTAGAGTCACACAC | | 678-729 |
| exon21 | TCTGGAATGCCAGAACTACAATCTTT | | TGGAATCCAGAGTGAGCTTTCATTT | | 1017-1068 |
| *FBXW7* | 4 | NM_0033632.2 | Tumor suppressor gene | |  |
| exon5 | GACAATGTTTAAAGGTGGTAGCTGTT | | TCTCTTTGATAGAGCTGGAGTGGA | | 247-287 |
| exon8 | GAAGAGTAAACTTACTTTGCCTGTGACT | | GCCTTCATTTTTCTCTTCACCAGTATTT | | 375-408 |
| exon9 | AGAAAGGGCCCAAATTCACCAATA | | TCATCACAAATGAGAGACAACATCATCA | | 435-473 |
| exon10 | AGTCTCTGGATCCCACACCTT | | TTGTTTTGTTTTTCTGTTTCTCCCTCTG | | 474-523 |
| exon11 | CACTGTCCTGTTTTGATATCCCAGATT | | ACTGTGTCTTTTGTAGTTTGATGGTATCC | | 553-604 |
| *PTEN* | 10 | NM_000314.4 | Tumor suppressor gene | |  |
| exon1 | AGCCATTTCCATCCTGCAGAAG | | TCCCACGTTCTAAGAGAGTGACA | | 1-27 |
| exon3 | AATTTCAAATGTTAGCTCATTTTTGTTAATGG | | CCTCACTCTAACAAGCAGATAACTTTCA | | 56-70 |
| exon6 | ACCCAGTTACCATAGCAATTTAGTGAAA | | CAAGTTCCGCCACTGAACAT | | 165-204 |
| exon7 | CTCAGCCGTTACCTGTGTGT | | ACCCTTATAATGTCTCACCAATGCC | | 251-267 |
| exon8 | TTTTTAGGACAAAATGTTTCACTTTTGGGT | | CGCTCTATACTGCAAATGCTATCGATT | | 276-299 |
| *SMAD4* | 18 | NM_005359.5 | Tumor suppressor gene | |  |
| exon3 | ATGGTCGTTTATTTTTCTAGGTGGCT | | TGAAAGCAAAGTCTACTTACCAATTCCA | | 86-139 |
| exon5 | ATGATGGTGAAGGATGAATATGTGCAT | | CTGGTAGCATTAGACTCAGATGGG | | 165-202 |
| exon6 | GCCCATCTTTATAGTTGTGCATTATCA | | GTAAGTAGCTGGCTGACCAGTAA | | 223-252 |
| exon8 | TGTTGTCTTTTCTTTAGGGCCTGTT | | GCGTTTCAATCACCACTAAATCAATCT | | 306-319 |
| exon9 | GGGAGGATGTTCTTTCCCATTTATT | | CAATACCTTGCTCTCTCAATGGCT | | 319-374 |
| exon10 | TTCCTAAGGTTGCACATAGGCAAA | | CCAGCTTCTCTGTCTAAGTAGTAACTCT | | 386-409 |
| exon11 | GTCTTTGATTTGCGTCAGTGTCA | | CTGCTCAAAGAAACTAATCAACTGAGT | | 445-483 |
| exon12 | CCTCTGATGTCTTCCAAATCTTTTCTG | | GTCTGCAATCGGCATGGTA | | 483-541 |
| *STK11* | 19 | NM_000455.4 | Tumor suppressor gene | |  |
| exon1 | GAGCTGATGTCGGTGGGTATG | | CGCAACTTCTTCTTCTTGAGGATCTT | | 23-77 |
| exon4 | GGCCTGGAGTACCTGCATA | | GGTCCGGCAGGTGTCGT | | 169-206 |
| exon6 | CTTTCTTCCCTCCCCTCGAAA | | CCCTACATTTCTGCACAAAAGCC | | 246-288 |
| exon8 | GAAGAAACATCCTCCGGCTGAA | | CTGGCCGAGTCAGCAGAG | | 318-370 |
| *TP53* | 17 | NM_000546.5 | Tumor suppressor gene | |  |
| exon2 | CCCTTCCAATGGATCCACTCA | | CCAGGGTTGGAAGTGTCTCAT | | 1-25 |
| exon4 | GCAACTGACCGTGCAAGTCA | | GTCCAGATGAAGCTCCCAGAAT | | 67-121 |
| exon5 | CGCCTCACAACCTCCGTCAT | | CTCCTTCCTCTTCCTACAGTACTCC | | 128-168 |
| exon6 | GGAGGTCAAATAAGCAGCAGGA | | AGCATCTTATCCGAGTGGAAGGA | | 200-224 |
| exon7 | GGCTCCTGACCTGGAGTCTT | | GGCCTGTGTTATCTCCTAGGTTG | | 226-257 |
| exon8 | GGCATAACTGCACCCTTGGT | | GCCTCTTGCTTCTCTTTTCCTATC | | 262-307 |
| exon10 | CTGGGCATCCTTGAGTTCCAA | | CTTTTGTACCGTCATAAAGTCAAACAAT | | 332-347 |

**^#^** All sequences are in the 5’ to 3’ orientation.
