# Supplementary material for: Development of a semi-conductor sequencing-based panel for genotyping of colon and lung cancer by the Onconetwork consortium
Source: BMC Cancer. 2015 Jan 31;15:26. doi: 10.1186/s12885-015-1015-5 (PMC4318366; doi:10.1186/s12885-015-1015-5)
Supplement: Additional file 3: Table S2. — Gene-panel version 2. [file 12885_2015_1015_MOESM3_ESM.docx]

**Supplementary table 2.** Gene-panel version 2.

| **Gene** | **Chr** | **RefSeq^#^** | **Oncogene / Tumor suppressor gene** | | **Codons** |
| --- | --- | --- | --- | --- | --- |
| *AKT1* | 14 | NM_005163.2 | Oncogene |  |  |
| exon3 | GCGCCACAGAGAAGTTGTTGA | | GGGTCTGACGGGTAGAGTGT | | 16-51 |
| *ALK* | 2 | NM_004304.3 | Oncogene |  |  |
| exon22 | AGGGTGTCTCTCTGTGGCTTTA | | GACTCTGTAGGCTGCAGTTCTC | | 1151-1172 |
| exon23 | TCTCTCGGAGGAAGGACTTGAG | | GCCCAGACTCAGCTCAGTTAAT | | 1172-1203 |
| *BRAF* | 7 | NM_004333.4 | Oncogene |  |  |
| exon11 | CATACTTACCATGCCACTTTCCCTT | | TTTCTTTTTCTGTTTGGCTTGACTTGA | | 439-472 |
| exon15 | CCACAAAATGGATCCAGACAACTGT | | GCTTGCTCTGATAGGAAAATGAGATCTA | | 582-610 |
| *CTNNB1* | 3 | NM_001904.3 | Oncogene |  |  |
| exon3 | ACTGTTTCGTATTTATAGCTGATTTGATGGA | | CCTCTTCCTCAGGATTGCCTTT | | 10-48 |
| *DDR2* | 1 | NM_006182.2 | Oncogene |  |  |
| exon5_1 | CAGTGAAAACTGTGGCAAGAACC | | AGGCTCCATCCCCTTCTTCTG | | 62-63 |
| exon5_2 | GTTTCTGCAGATTGACTTGCACA | | CTTCTTCCTACCTGTTTCCCATGAC | | 93-134 |
| exon8 | CCTGAGCAGCATGACAGAAG | | AACATGATCTCAATGTAGCCATTGGT | | 228-264 |
| exon12 | CTCGGAGGATGCTGGATGATG | | AGTTTTCGTATCAGCCTGGATGG | | 440-483 |
| exon13 | CTCCAAAGACACTCCACGGAA | | GACGGCAGGCACTGAGTA | | 502-537 |
| exon14 | CAGCAAGAGTACTGAGACATCTTCA | | TCTGCTCGGAGCATTTTCACA | | 577-605 |
| exon15 | TGTCTGTATCCTCCCAAGGAATGA | | CTCGTGGCGGGAAAGAAAC | | 622-663 |
| exon17 | GGTGTTGTTGTGCACAGGTTAT | | CTGTTCATCTGACAGCTGGGAA | | 762-788 |
| *EGFR* | 7 | NM_005228.3 | Oncogene |  |  |
| exon12 | TCAGGAAACAAAAATTTGTGCTATGCAA | | GGACCCATTAGAACCAACTCCAT | | 473-499 |
| exon18 | TGTGGAGCCTCTTACACCCA | | GTGCCAGGGACCTTACCTTATAC | | 695-725 |
| exon19 | ACGTCTTCCTTCTCTCTCTGTCA | | CTGAGGTTCAGAGCCATGGA | | 729-761 |
| exon20_1 | CATGCGAAGCCACACTGAC | | CGGACATAGTCCAGGAGGCA | | 762-796 |
| exon20_2 | GACTATGTCCGGGAACACAAAGA | | CCCCATGGCAAACTCTTGCTA | | 808-823 |
| exon21 | CGCAGCATGTCAAGATCACAGAT | | GCATGTGTTAAACAATACAGCTAGTG | | 856-875 |
| *ERBB2* | 17 | NM_004448.2 | Oncogene |  |  |
| exon19 | GAATGTGAAAATTCCAGTGGCCATC | | GTCATATCTCCCCAAACCCCAAT | | 753-769 |
| exon20 | GGGTGTGTGGTCTCCCATAC | | GCCATAGGGCATAAGCTGTGTC | | 770-796 |
| exon21 | GGATGAGCTACCTGGAGGATGT | | CCTTGGTCCTTCACCTAACCTTG | | 840-881 |
| *ERBB4* | 2 | NM_005235.2 | Oncogene |  |  |
| exon3 | GCCTTAGAGTGTTCCTCAATGTAACAA | | GAAACTTTGGACTTCAAGAACTTGGAT | | 127-141 |
| exon4 | CATCGCCACATAGGGTAGAACATTT | | CAGACACCATTCATTGGCAAGATATTG | | 167-186 |
| exon6 | CCTGAATCAAATAGGGAAGGAAAGGA | | GGCAGATGCTACGGACCTTA | | 226-247 |
| exon7 | TCTGTTACTTACGTGGACATTTCTTGAC | | CAGGCCTGCATGAATTTCAATGA | | 255-289 |
| exon8 | GGCAAATGTCAGTGCAAGGTTTA | | TGTTTTGAGCTTGTTTGCTGAATGT | | 295-322 |
| exon9 | ACCCATGAATACCAGTGACTAGAAAGA | | CTCAATCCCCTAACTCTGAGTCTTG | | 333-366 |
| exon15 | GCCAGCAAGAATGCTTACCCTT | | GGGTCCTGACAACTGTACAAAGT | | 580-622 |
| exon23 | CATTTGACCATGACCATGTAAACGTC | | GGAACTGATGACCTTTGGAGGAA | | 919-947 |
| *FGFR1* | 8 | NM_023110.2 | Oncogene |  |  |
| exon4 | ACCCAAAGGGCAGTAAGATAGGAA | | GGTCCCTAGGAGGAACCTCA | | 120-150 |
| exon7 | GGTCACTGTACACCTTACACATGAA | | CCCTCTTTAGCCATGGCAAGG | | 249-174 |
| *FGFR2* | 10 | NM_000141.4 | Oncogene |  |  |
| exon7_1 | CATCACTGTAAACCTTGCAGACAAAC | | TGGTCTCTCATTCTCCCATCCC | | 250-275 |
| exon7_2 | CATCCTCTCTCAACTCCAACAGG | | AGTGGATCAAGCACGTGGAAAA | | 297-313 |
| exon9 | GCTTCTTGGTCGTGTTCTTCATT | | CTCCTCCTGTGATCTGCAATCT | | 363-400 |
| exon12 | TGGAAGCCCAGCCATTTCTAAA | | GATGATGAAGATGATTGGGAAACACAAG | | 547-559 |
| *FGFR3* | 4 | NM_000142.4 | Oncogene |  |  |
| exon7 | GCCCCTGAGCGTCATCT | | GGGCTGTGCGTCACTGTA | | 247-277 |
| exon9 | GAGCTGGTGGAGGCTGA | | GGAGCCCAGGCCTTTCTT | | 368-402 |
| exon14 | GTGACCGAGGACAACGTGAT | | GCGTCCTACTGGCATGACC | | 632-653 |
| exon16 | CTCTGGGAGATCTTCACGCT | | CCACTCACAGGTCGTGTGT | | 691-719 |
| exon18 | CGCCTTTCGAGCAGTACTCC | | GCTAGGGACCCCTCACATTGT | | 772-806 |
| *KRAS* | 12 | NM_004985.3 | Oncogene |  |  |
| exon2 | CAAAGAATGGTCCTGCACCAGTAATAT | | AGGCCTGCTGAAAATGACTGAATATAA | | 6-37 |
| exon3 | TCCTCATGTACTGGTCCCTCATT | | GTAAAAGGTGCACTGTAATAATCCAGACT | | 38-66 |
| exon4 | CAGATCTGTATTTATTTCAGTGTTACTTACCT | | GACTCTGAAGATGTACCTATGGTCCTA | | 114-149 |
| *MAP2K1* | 15 | NM_002755.3 | Oncogene |  |  |
| exon2 | GCTGGAGGAGCTAGAGCTTGAT | | GGCTTGTGGGAGACCTTGAAC | | 44-82 |
| *MET* | 7 | NM_001127500.1 | Oncogene |  |  |
| exon2_1 | CTGACATACAGTCGGAGGTTCAC | | AGAAGTTGATGAACCGGTCCTTT | | 160-188 |
| exon2_2 | CAAATAGGAGCCAGCCTGAATGAT | | GGAGACATCTCACATTGTTTTTGTTGA | | 340-377 |
| exon14 | CCCATGATAGCCGTCTTTAACAAG | | CGGTAGTCTACAGATTCATTTGAAACCAT | | 981-1012 |
| exon16 | TGTTACGCAGTGCTAACCAAGTT | | GCAAACCACAAAAGTATACTCCATGGT | | 1105-1132 |
| exon19 | GCTGATTTTGGTCTTGCCAGAG | | TCTGACTTGGTGGTAAACTTTTGAGTT | | 1246-1274 |
| *NOTCH1* | 9 | NM_017617.3 | Oncogene |  |  |
| exon26 | CACGCTTGAAGACCACGTTG | | GGACTGTGCGGAGCATGTA | | 1566-1602 |
| exon27 | ACACACTGCCGGTTGTCAA | | CCTCACCATGTCCTGACTGTG | | 1674-1679 |
| *NRAS* | 1 | NM_002524.3 | Oncogene |  |  |
| exon2 | CCTCACCTCTATGGTGGGATCATAT | | GTTCTTGCTGGTGTGAAATGACTG | | 3-31 |
| exon3 | TTCGCCTGTCCTCATGTATTGG | | CACCCCCAGGATTCTTACAGAAAA | | 43-69 |
| *PIK3CA* | 3 | NM_006218.2 | Oncogene |  |  |
| exon10 | CAGAGTAACAGACTAGCTAGAGACAATGA | | GCACTTACCTGTGACTCCATAGAAA | | 523-549 |
| exon14 | CACGATTCTTTTAGATCTGAGATGCACA | | CCTTTTGTGTTTCATCCTTCTTCTCCTG | | 677-720 |
| exon21_1 | TGGAATGCCAGAACTACAATCTTTTGAT | | GTGGAAGATCCAATCCATTTTTGTTGTC | | 1018-1051 |
| exon21-2 | TGGATCTTCCACACAATTAAACAGCAT | | TGCTGTTCATGGATTGTGCAATTC | | 1067-1069 |
| *FBXW7* | 4 | NM_0033632.2 | Tumor suppressor gene | |  |
| exon5 | TGACAATGTTTAAAGGTGGTAGCTGTT | | ACTCATTGATAGTTGTGAACCAACACA | | 265-287 |
| exon8 | CCTGTGACTGCTGACCAAACTTTTA | | CACATCTTTCTTATAGGTGCTGAAAGG | | 379-402 |
| exon9 | CCCAACCATGACAAGATTTTCCC | | GGTCATCACAAATGAGAGACAACATCA | | 434-473 |
| exon10 | ACTAACAACCCTCCTGCCATCATA | | TCTGCAGAGTTGTTAGCGGTT | | 478-508 |
| exon11 | GTAGAATCTGCATTCCCAGAGACAA | | TCTCTTGATACATCAATCCGTGTTTGG | | 567-593 |
| *PTEN* | 10 | NM_000314.4 | Tumor suppressor gene | |  |
| exon1 | GCCATCTCTCTCCTCCTTTTTCTT | | GCCGCAGAAATGGATACAGGTC | | 1-25 |
| exon3 | TGTTAATGGTGGCTTTTTGTTTGTTTGT | | TCTACCTCACTCTAACAAGCAGATAACT | | 56-70 |
| exon6 | GGCTACGACCCAGTTACCATAG | | TGCCACTGGTCTATAATCCAGATGAT | | 165-183 |
| exon7_1 | TGAGATCAAGATTGCAGATACAGAATCC | | ACCTTTAGCTGGCAGACCAC | | 212-215 |
| exon7_2 | AGGTGAAGATATATTCCTCCAATTCAGGAC | | TTGGATATTTCTCCCAATGAAAGTAAAGTAC | | 231-267 |
| exon8_1 | CACTTTTGGGTAAATACATTCTTCATACCAGGA | | CACGCTCTATACTGCAAATGCTATCGA | | 283-299 |
| exon8_2 | GCAGTATAGAGCGTGCAGATAATGA | | CATCACATACATACAAGTCAACAACCC | | 313-342 |
| *SMAD4* | 18 | NM_005359.5 | Tumor suppressor gene | |  |
| exon3 | CTCATGTGATCTATGCCCGTCT | | AGTCTACTTACCAATTCCAGGTGATACA | | 99-136 |
| exon5 | ATGGTGAAGGATGAATATGTGCATGA | | GCTGGTAGCATTAGACTCAGATGG | | 166-202 |
| exon6 | GTGAAGGACTGTTGCAGATAGCAT | | AAGGCCCACATGGGTTAATTTG | | 242-263 |
| exon8 | TTTCTTTAGGGCCTGTTCACAATGA | | CTGAGAAGTGACCCCATAATTCCATT | | 308-319 |
| exon9 | GCTCCTGAGTATTGGTGTTCCAT | | CCTGTGGACATTGGAGAGTTGA | | 327-365 |
| exon10 | TGTAATTTCTTTTTTCTTCCTAAGGTTGCACATAG | | ACTTGGGTAGATCTTATGAACAGCAT | | 384-423 |
| exon11 | AGGTCTTTGATTTGCGTCAGTGT | | GCTGGAGCTATTCCACCTACTG | | 444-473 |
| exon12 | GCTGCTGGAATTGGTGTTGATG | | AGTACTTCGTCTAGGAGCTGGAG | | 494-532 |
| *STK11* | 19 | NM_000455.4 | Tumor suppressor gene | |  |
| exon1 | GAGCTGATGTCGGTGGGTAT | | CTCCGAGTCCAGCACCTC | | 23-64 |
| exon4 | CCGGTGGCACCCTCAAA | | CTGGTCCGGCAGGTGTC | | 192-199 |
| exon5 | AACATCACCACGGGTCTGTAC | | GATGAGGCTCCCACCTTTCAG | | 200-207 |
| exon6 | GAAGAAACATCCTCCGGCTGAA | | ACCGTGAAGTCCTGAGTGTAGA | | 254-285 |
| *TP53* | 17 | NM_000546.5 | Tumor suppressor gene | |  |
| exon2 | TCCACTCACAGTTTCCATAGGTCT | | GTTGGAAGTGTCTCATGCTGGAT | | 1-20 |
| exon4 | GGCTGTCCCAGAATGCAAGAA | | GATGAAGCTCCCAGAATGCCA | | 68-112 |
| exon5_1 | TGCACAGGGCAGGTCTTG | | CCGTCTTCCAGTTGCTTTATCTGT | | 126-138 |
| exon5_2 | ACCAGCCCTGTCGTCTCT | | GTGCAGCTGTGGGTTGATTC | | 150-187 |
| exon6 | CCAGTTGCAAACCAGACCTCA | | AGGCCTCTGATTCCTCACTGAT | | 187-223 |
| exon7 | GGCTCCTGACCTGGAGTCTT | | CTCATCTTGGGCCTGTGTTATCTC | | 225-257 |
| exon8 | CGCTTCTTGTCCTGCTTGCT | | TTCTCTTTTCCTATCCTGAGTAGTGGT | | 263-307 |
| exon10 | GGAAGGGGCTGAGGTCACT | | CCCCTCCTCTGTTGCTGC | | 332-367 |

**^#^** All sequences are in the 5’ to 3’ orientation.
