# Supplementary material for: Development of a semi-conductor sequencing-based panel for genotyping of colon and lung cancer by the Onconetwork consortium
Source: BMC Cancer. 2015 Jan 31;15:26. doi: 10.1186/s12885-015-1015-5 (PMC4318366; doi:10.1186/s12885-015-1015-5)
Supplement: Additional file 4: Table S3. — Known mutations present in the 90 samples that were analyzed during phase 3 of the panel validation. [file 12885_2015_1015_MOESM4_ESM.docx]

| **Mutation** | **Unique samples** | **Identified** |
| --- | --- | --- |
| KRAS: p.Gly12Ala | 2 | Yes |
| KRAS: p.Gly12Arg | 1 | Yes |
| KRAS: p.Gly12Asp | 7 | Yes |
| KRAS: p.Gly12Cys; STK11:p.Asp176Val | 1 | Yes |
| KRAS: p.Gly12Cys | 3 | Yes |
| KRAS: p.Gly12Phe | 1 | No, no data |
| KRAS: p.Gly12Ser | 1 | Yes |
| KRAS: p.Gly12Val | 1 | Yes |
| KRAS: p.Gly13Asp | 8 | Yes |
| KRAS: p.Gly13Cys | 1 | Yes |
| KRAS: p.Thr58Ile | 1 | Yes |
| ERBB2: p.Gly776delinsValCys | 1 | Yes |
| BRAF: p.Gly469Val | 1 | Yes |
| BRAF: p.Val600Glu | 7 | Yes |
| EGFR: p.Asn842His; BRAF: p.Val600Glu | 1 | Yes |
| EGFR: p.Ala767_Val769dup | 1 | Yes |
| EGFR: p.Leu747_Thr751del | 1 | Yes |
| EGFR: p.Glu746_Ser752delinsVal | 1 | Yes |
| EGFR: p.Glu746_Ala750del | 4 | Yes |
| EGFR: p.Glu746_Ala750del; p.Thr790Met | 1 | Yes |
| EGFR: p.Glu746_Thr751delinsAla | 1 | Yes |
| EGFR: p.Gly719Ala; p.Gln701Leu | 1 | Yes |
| EGFR: p.Leu747_Ala750delinsPro | 1 | Yes |
| EGFR: p.Leu858Arg | 5 | Yes |
| EGFR: p.Leu861Gln | 1 | Yes |

**Supplementary Table 3.** Known mutations present in the 90 samples that were analyzed during phase 3 of the panel validation.
