# Supplementary material for: Development of a semi-conductor sequencing-based panel for genotyping of colon and lung cancer by the Onconetwork consortium
Source: BMC Cancer. 2015 Jan 31;15:26. doi: 10.1186/s12885-015-1015-5 (PMC4318366; doi:10.1186/s12885-015-1015-5)
Supplement: Additional file 6: Table S5. — Approximate indication of hands on time and costs of Sanger sequencing and Next Generation Sequencing of the genetic regions covered by the described gene panel. [file 12885_2015_1015_MOESM6_ESM.docx]

**Supplementary table 5.** Approximate indication of hands on time and costs of Sanger sequencing and Next Generation Sequencing of the genetic regions covered by the described gene panel.

|  | Sanger sequencing | NGS using IonPGM |
| --- | --- | --- |
| Total time*/hands on time (1 amplicon or library) | 7h/75’ | 3 days/6.5h |
| Cost/amplicon | €30 | NA |
|  |  |  |
| **10 amplicons (BRAF, EGFR, KRAS, NRAS)** |  |  |
| Costs | €300 | €250 |
| Amount of DNA | 100 ng | 10 ng |
| Total time/hands on time for 1 sample | 9h/2.5h | 3 days/6.5h |
| Total time/hands on time for 8 samples | 21h (3 days)/4h | 3 days/9h |
| **90 amplicons (entire gene-panel)** |  |  |
| Costs | €2.700 | €250 |
| Amount of DNA | 900 ng | 10 ng |
| Total time/hands on time for 1 sample | 16h/5h | 3 days/6.5h |
| Total time/hands on time for 8 samples | 60h (5 days)/14h | 3 days/9h |
|  |  |  |

*Time estimation for Sanger sequencing based on a 16 capillaries sequencer. Costs may vary between labs and countries. A more extensive comparison has been published by *Endris, et al.* (18).
